# Radiology of Trauma

**Published:** 1992-03

**Authors:** Roger Laitt


**RADIOLOGY OF TRAUMA**
**by S. Sclafani**
Gower. $59.95

The paramount role of radiology in the management of trauma has expanded with the development of new imaging modalities. A new text on the radiology of trauma is therefore welcome. Professor Sciafani has produced such a book, a glossy publication of 150 pages divided into two sections, skeletal and non-skeletal, trauma, that is well indexed and easy to use.

The first section concerns the plain radiographic assessment of fractures with six chapters each concerned with a particular region of the body. There are anatomical drawings at the beginning of each chapter but these would be of more value accompanied by corresponding radiographs. The majority of common fractures are demonstrated and the use of CT in further evaluation, particularly of spinal and pelvic trauma, is emphasised. Unfortunately, the standard of reproduction of the many figures is poor and accompanying line drawings add little. A chapter of normal variants that simulate fractures, a minefield for junior radiologists and casualty officers, would have been of value.

The second section on non-skeletal trauma is of greater interest. There are five chapters including useful but brief accounts of chest, abdominal and urinary tract injury. The value of CT is again made clear but the use of ultrasound is underplayed, particularly in the assessment of abdominal trauma.

It is unclear as to which audience this book is aimed. It is too brief to be of value to radiology trainees but may be useful in a busy casualty department.

Roger Laitt

